# Comprehensive transcriptional and functional analyses of melatonin synthesis genes in cassava reveal their novel role in hypersensitive-like cell death

**DOI:** 10.1038/srep35029

**Published:** 2016-10-14

**Authors:** Yunxie Wei, Wei Hu, Qiannan Wang, Wei Liu, Chunjie Wu, Hongqiu Zeng, Yu Yan, Xiaolin Li, Chaozu He, Haitao Shi

**Affiliations:** 1 Hainan Key Laboratory for Sustainable Utilization of Tropical Bioresources, College of Agriculture, Hainan University, Haikou, 570228, China; 2 Key Laboratory of Biology and Genetic Resources of Tropical Crops, Institute of Tropical Bioscience and Biotechnology, Chinese Academy of Tropical Agricultural Sciences, Xueyuan Road 4, Haikou, Hainan province, 571101, China

## Abstract

Melatonin is a widely known hormone in animals. Since melatonin was discovered in plants, more and more studies highlight its involvement in a wide range of physiological processes including plant development and stress responses. Many advances have been made in the terms of melatonin-mediated abiotic stress resistance and innate immunity in plants, focusing on model plants such as rice and *Arabidopsis*. In this study, 7 melatonin synthesis genes were systematically analyzed in cassava. Quantitative real-time PCR showed that all these genes were commonly regulated by melatonin, flg22, *Xanthomonas axonopodis* pv *manihotis* (*Xam*) and hydrogen peroxide (H_2_O_2_). Transient expression in *Nicotiana benthamiana* revealed the subcellular locations and possible roles of these melatonin synthesis genes. Notably, we highlight novel roles of these genes in hypersensitive-like cell death, as confirmed by the results of several physiological parameters. Moreover, transient expression of these genes had significant effects on the transcripts of reactive oxygen species (ROS) accumulation and defense-related genes, and triggered the burst of callose depositions and papillae-associated plant defense, indicating the possible role of them in plant innate immunity. Taken together, this study reveals the comprehensive transcripts and putative roles of melatonin synthesis genes as well as melatonin in immune responses in cassava.

In 1958, N-acetyl-5-methoxytryptamine (melatonin) was first discovered in the pineal gland of cow, thereafter melatonin was widely identified in multiple animals^1^. In 1995, melatonin was identified by two research groups in plants^2^,^3^. So far, using gas chromatography/mass spectrometry (GC/MS) and radioimmunoassay, melatonin has been identified in more and more plant species with different levels, including multiple edible plants (banana, cucumber, apple, coffee, corn)^4^, lupin^5^, tomato^6^,^7^,^8^,^9^, rice^9^,^10^,^11^,^12^,^13^,^14^,^15^, sweet cherry^16^, *Arabidopsis*^17^,^18^, bermudagrass^19^, etc. Moreover, it was found that different plant species, plant organs, plant stages, plant location and treatments had significant effects on endogenous melatonin levels^19^,^20^,^21^,^22^. The wide distribution of melatonin in plants especially in popular beverages and crops makes people can daily take in melatonin from the related products, and this may be benefit to human for the well-known beneficial effects of melatonin on human health^23^,^24^,^25^.

To date, melatonin biosynthetic and metabolic pathway in plants have been revealed. Melatonin biosynthesis begins from tryptophan, the same as it does in animals^19^,^20^,^21^,^22^,^23^,^24^,^25^,^26^,^27^,^28^. Four sequential enzymatic steps are involved in melatonin biosynthesis in plants, including tryptophan decarboxylase (TDC), tryptamine 5-hydroxylase (T5H), serotonin *N*-acetyltransferase (SNAT) and *N*-aceylserotonin *O*-methyltransferase (ASMT)^29^,^30^,^31^,^32^,^33^,^34^. Moreover, melatonin 2-hydroxylase (M2H) is also responsible for melatonin metabolism, by converting melatonin to 2-hydroxymelatonin^35^. Genetic modulation by overexpression or knockdown of the transcripts of the above genes responsible for melatonin biosynthesis from animals and plants, including *OsTDC*, *OsASMT*, *C. reinhardtii AANAT*, ovine *AANAT* and *HIOMT*, found that the underlying gene expressions are directly associated with endogenous melatonin production^19^,^20^,^21^,^22^,^23^,^24^,^25^,^26^,^27^,^34^,^36^,^37^,^38^.

Through exogenous application of melatonin or using transgenic plants with affected melatonin levels, previous studies have shown that melatonin plays multiple roles in plants, including senescence^39^,^40^,^41^,^42^,^43^, root development^44^,^45^,^46^, floral transition^47^, fruit ripening^48^, postharvest physiological deterioration^49^,^50^, seed germination^51^, plant abiotic and biotic stresses^52^,^53^,^54^,^55^,^56^,^57^,^58^,^59^,^60^,^61^,^62^,^63^,^64^,^65^,^66^,^67^,^68^,^69^,^70^,^71^. In recent years, many advances have been made in the terms of melatonin-mediated abiotic stress resistance and innate immunity in plants, including salt, drought, cold, heat and oxidative stresses and biotic stress such as pathogen infection^19^,^20^,^21^,^22^,^23^,^24^,^25^,^26^,^27^.

Cassava (*Manihot esculenta*) can be cultivated under adverse environmental (drought and heat) and nutrient-limited conditions (low phosphorus and low nitrogen), together with its high photosynthetic efficiencies and starch enrichment, making it be considered as an energy crop and important tropical crop^72^,^73^,^74^,^75^. So far, only two manuscripts reported the *in vivo* role of melatonin in cassava^49^,^50^. Melatonin delays the postharvest physiological deterioration of cassava storage root, through modulation of ROS metabolism, starch metabolism, calcium signaling and mitogen-activated protein kinase (MAPK) cascades^49^,^50^. To extend our understanding of melatonin in cassava stress responses, it is essential to reveal the function of melatonin synthesis genes in cassava. In this study, 7 melatonin synthesis genes were cloned and functionally analyzed, especially their possible involvement in immune response. The results may help us in understanding the putative roles of these genes as well as melatonin in immune responses in cassava.

## Results

### Isolation and expression profiles of melatonin synthesis genes in cassava

**Figure 1:**
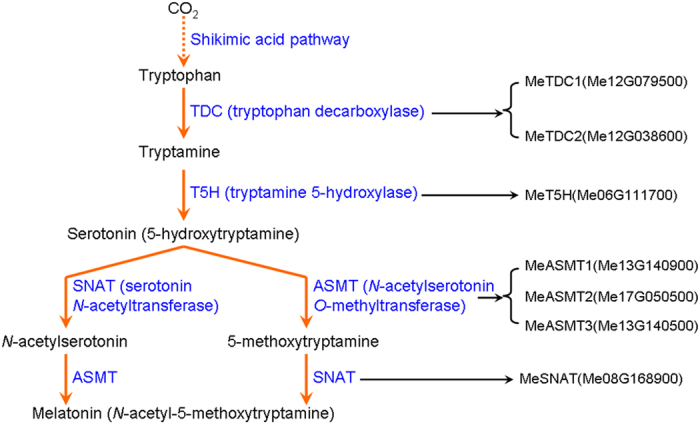
The melatonin synthesis genes responsible for melatonin synthesis in cassava.

Based on previous studies^29^,^30^,^31^,^32^,^33^,^34^,^76^, the enzymes responsible for melatonin biosynthesis in plants have been largely revealed. Through genome-wide identification in cassava (*Manihot esculenta*) annotation database at Phytozome v10.3 (http://www.phytozome.net/cassava.php), 7 cassava genes were identified as candidate genes, including 2 *MeTDCs*, 1 *MeT5H*, 1 *MeSNAT* and 3 *MeASMTs* (Fig. 1). Moreover, the molecular weight (MW) and theoretical pI of these proteins were analyzed by ProtParam software (http://web.expasy.org/protparam). Then the detailed information of these genes were listed in Table 1, including the locus name, chromosome location, the lengths of peptide, CDS, cDNA and genome of these genes.

**Figure 2:**
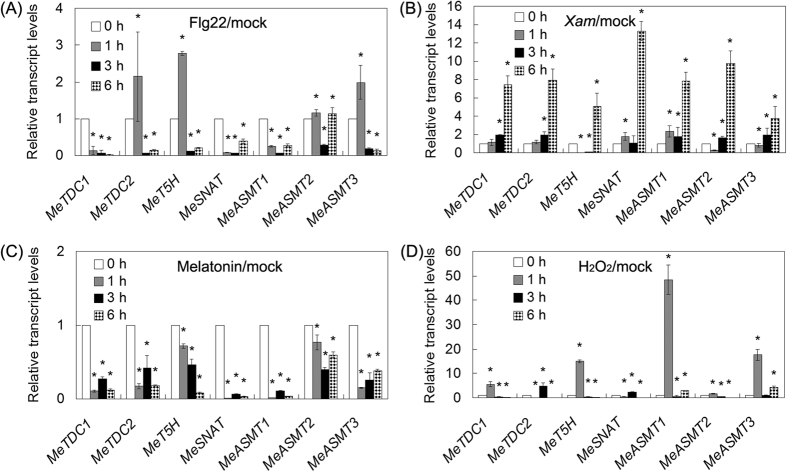
The expression profiles of melatonin synthesis genes in response to flg22 (**A**), *Xam* (**B**), melatonin (**C**) and H2O2 (**D**). For the assays, about 30-day-old cassava leaves were treated with water (mock), or 200 μM melatonin, or 10 μM flg22, or *Xam* infection, or 5 mM H_2_O_2_ for 0, 1, 3 and 6 h. The gene transcripts of all genes at 0 h of mock treatment were normalized as 1.0. Asterisk symbols (*) were shown as significant difference at *p* < 0.05.

Through quantitative real-time PCR, we found that the transcript levels of 7 melatonin synthesis genes (*MeTDC1*, *MeTDC2*, *MeT5H*, *MeSNAT*, *MeASMT1*, *MeASMT2* and *MeASMT3*) were significantly affected after treatment with flg22, *Xanthomonas axonopodis* pv *manihotis* (*Xam*), melatonin or hydrogen peroxide (H_2_O_2_) for 1, 3 and 6 h (Fig. 2A–D). Notably, all these gene transcripts were commonly down-regulated by exogenous melatonin treatment, but were largely up-regulated by *Xam* and H_2_O_2_ at least in one time-point (Fig. 2B–D). However, the transcript levels of these genes were differentially regulated by flg22 treatment (Fig. 2A). Generally, the transcripts levels of these genes were consistent with endogenous melatonin levels in cassava response to flg22, *Xam* and H_2_O_2_ treatments (Supplemental Fig. S1). The common expression profile of 7 melatonin synthesis genes in response to flg22, *Xam*, melatonin or H_2_O_2_ treatments, indicates the possible role of them as well as melatonin in immune response and reactive oxygen species (ROS) signaling in cassava. Thus, melatonin synthesis pathways may play some roles in cassava immune response.

### Subcellular localization of melatonin synthesis genes in cassava

**Figure 3:**
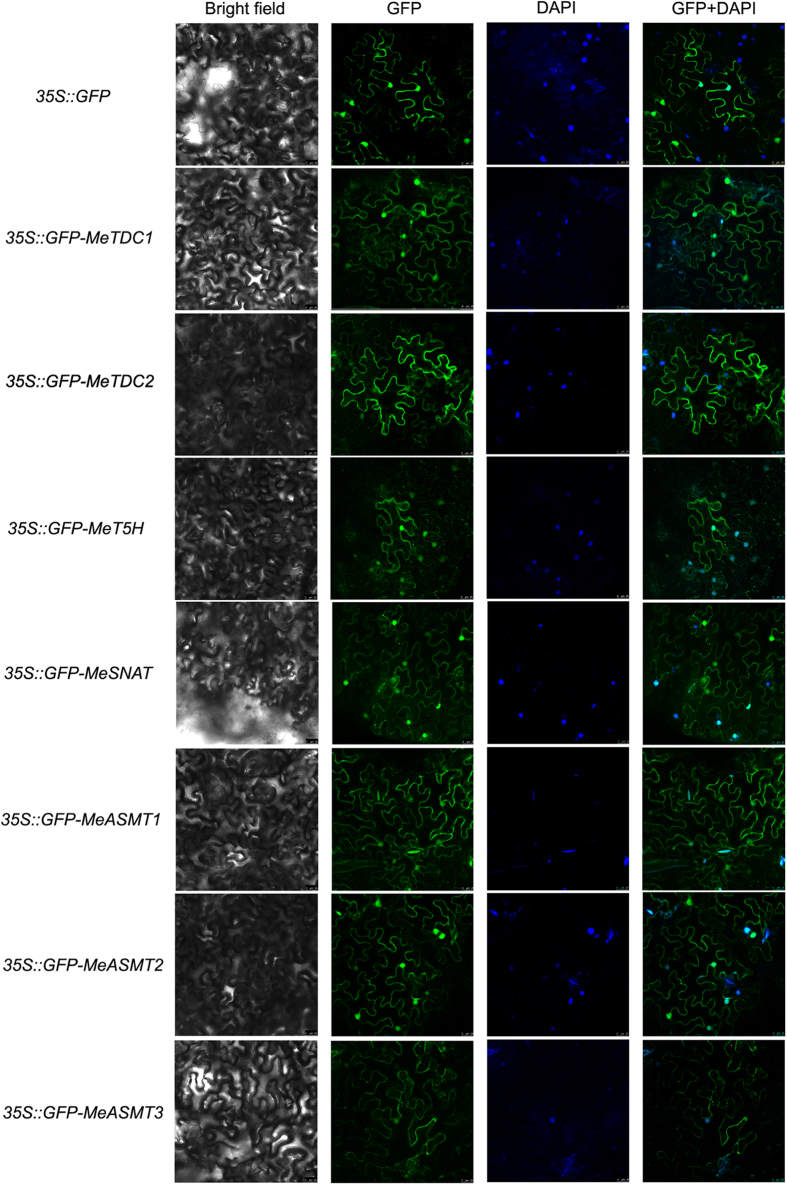
Subcellular localization of melatonin synthesis genes in cassava. Transient expression of GV3101 cell culture harbouring the *35S::GFP*, *35S::GFP-MeTDC1*, *35S::GFP-MeTDC2*, *35S::GFP-MeSNAT*, *35S::GFP-MeT5H*, *35S::GFP-MeASMT1*, *35S::GFP-MeASMT2* and *35S::GFP-MeASMT3* plasmids in tobacco leaves. Cell nuclei was stained by 1 μg/ml 4′,6-diamidino-2-phenylindole (DAPI). Bar = 25 μm.

To investigate the subcellular location of 7 melatonin synthesis genes in cassava, the CDS of these genes were fused in the in-frame with green fluorescent protein (GFP) reporter gene and express in tobacco (*N. benthamiana*) leaves. After 2 days post infiltration (dpi), we found that the fusion proteins of GFP and 7 melatonin synthesis proteins (MeTDC1, MeTDC2, MeT5H, MeSNAT, MeASMT1, MeASMT2 and MeASMT3) exhibited obvious green signals in both nucleus and cell membrane in the leaf cells (Fig. 3).

### Transient expression of melatonin synthesis genes triggers hypersensitive response-like cell death

**Figure 4:**
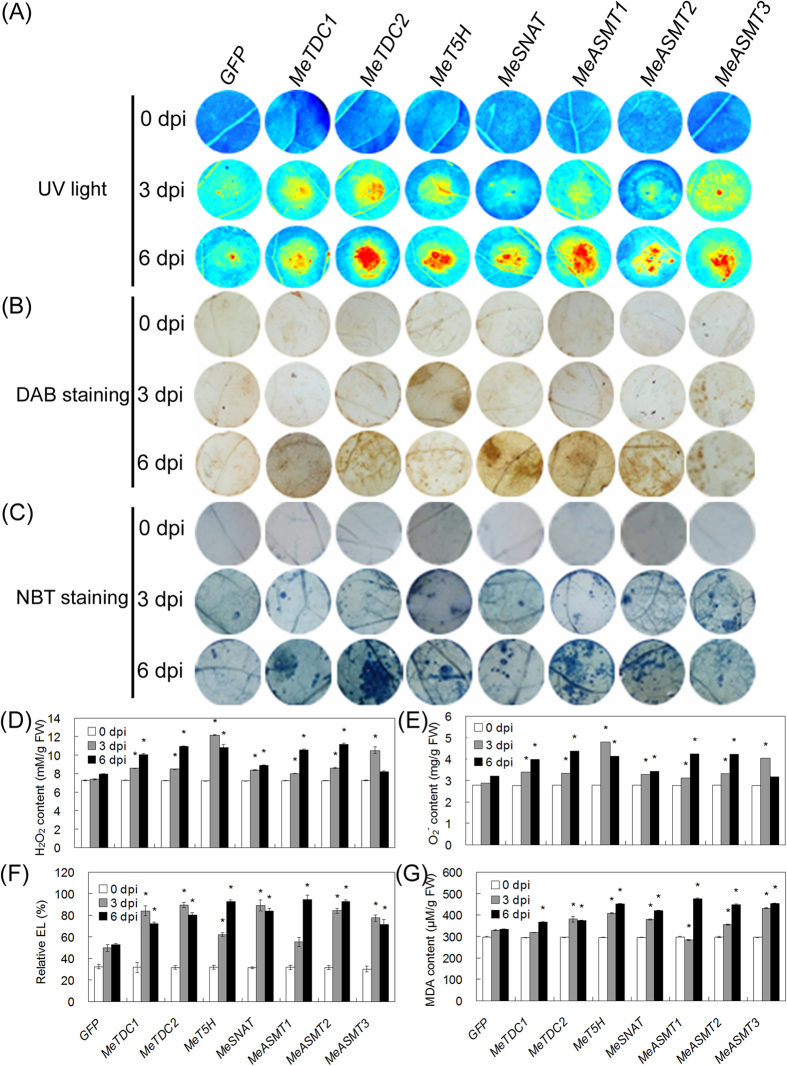
Transient expression of melatonin synthesis genes in tobacco leaves on hypersensitive response-like cell death. (**A**) Symptoms of cell death in leaves were visualized under UV light using the ChemiDoc Imaging System. (**B,C**) DAB staining for endogenous H_2_O_2_ level (**B**) and NBT staining for endogenous O_2_•^−^ level (**C**) in tobacco leaf discs expressing different plasmids. (**D–G**) Quantification of H_2_O_2_ (D), O_2_•^−^ (**E**), EL (**F**) and MDA (**G**) contents in leaf discs expressing different plasmids. Asterisk symbols (*) were shown as significant difference at *p* < 0.05.

Interestingly, we observed that the transient expression of all these genes resulted in obvious cell death and hypersentive response (HR) symptoms after infiltration in tobacco (*N. benthamiana*) leaves in comparison to the overexpression of GFP (control) (Fig. 4A). HR is a common type of programmed cell death (PCD) as well as a marker feature of plant immune response, which are largely related to ROS accumulation^77^,^78^. In this study, tobacco leaves expressing melatonin synthesis genes exhibited significantly higher levels of H_2_O_2_ and superoxide radical (O_2_•^−^) than those from leaves expressing GFP alone, as evidenced by DAB, NBT staining (Fig. 4B,C) and the quantifications of endogenous H_2_O_2_ and O_2_•^−^ (Fig. 4D,E). Moreover, the electrolyte leakage (EL) of leaf discs from tobacco leaves expressing these genes was significantly higher than those from leaves expressing GFP (Fig. 4F), indicating the significant increase of ion leakage triggered by these genes transient expression. Consistently, malondialdehyde (MDA), which is a lipid peroxidation caused by intracellular ROS accumulation, displayed high level in tobacco leaves expressing these genes than GFP (Fig. 4G). These results indicate the *in vivo* roles of melatonin synthesis genes in HR, immune response and underlying ROS modulation.

### Modulation of melatonin synthesis genes expression regulates melatonin content

**Figure 5:**
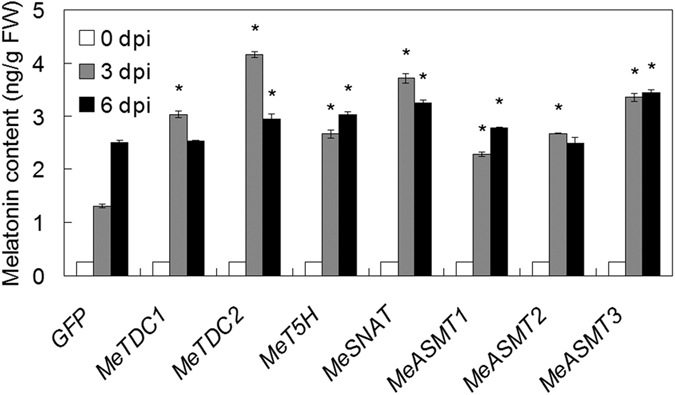
The endogenous melatonin levels induced by the expression of melatonin synthesis genes expression. Asterisk symbols (*) were shown as significant difference at *p* < 0.05.

To reveal the possible relationship between melatonin synthesis genes-regulated HR and endogenous melatonin level, we investigate the effect of these genes transient expression on melatonin level. Through quantification by ELISA, we found that tobacco leaves expressing melatonin synthesis genes exhibited significantly higher levels of endogenous melatonin than those from leaves expressing GFP alone (Fig. 5), suggesting the possible involvement of melatonin in these genes -mediated HR and immune response. Although MeT5H gene is missing at least 70 aa at the N-terminus when compared to other MeT5H homologs such as OsT5H^55^, it had significant effect on melatonin synthesis, indicating the difference of T5H in different plant species.

### Modulation of melatonin synthesis genes expression triggers immune response

**Figure 6:**
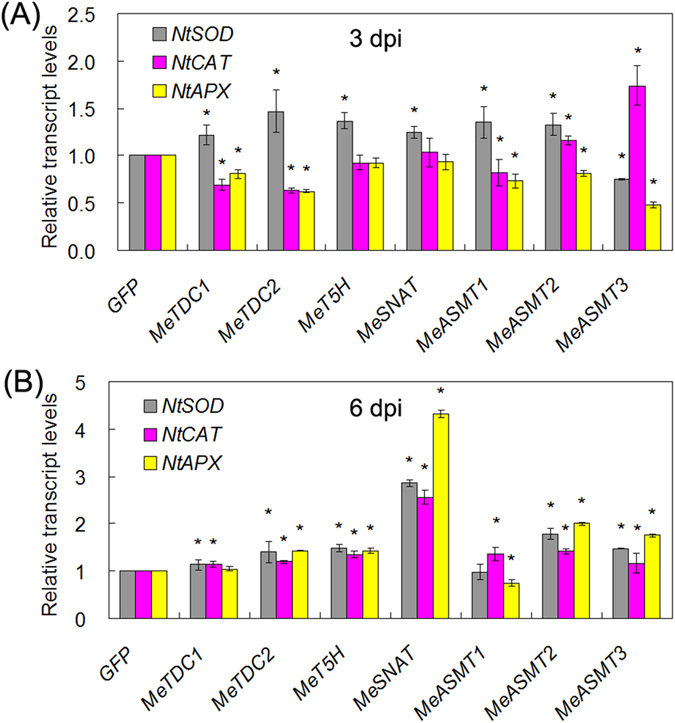
Modulation of melatonin synthesis genes expression regulates the transcripts of ROS-related genes. The gene expression was assayed at 3 dpi (**A**) and 6 dpi (**B**). The transcript levels of every gene in mock (GFP) transformed leaves were set as 1. Asterisk symbols (*) were shown as significant difference at *p* < 0.05.

**Figure 7:**
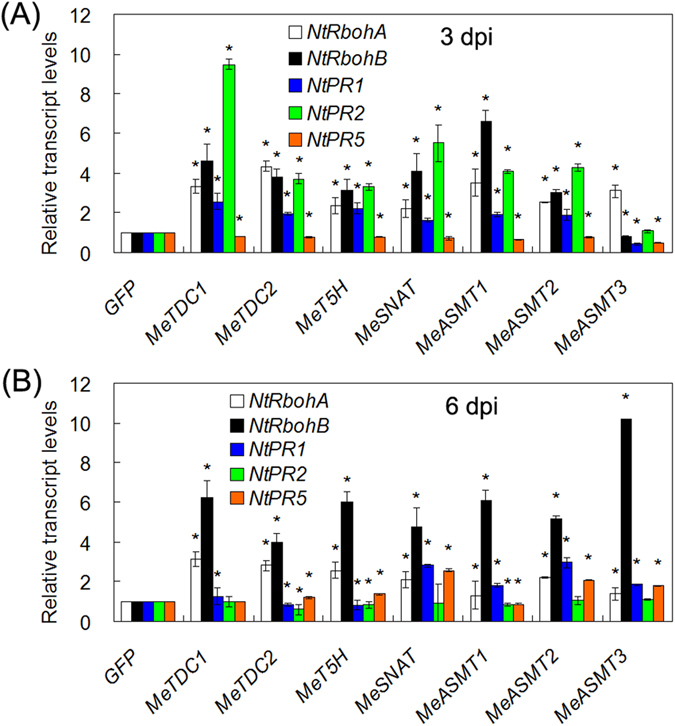
Modulation of melatonin synthesis genes expression regulates the transcripts of several genes in plant immunity. The gene expression was assayed at 3 dpi (**A**) and 6 dpi (**B**). The transcript levels of every gene in mock (GFP) transformed leaves were set as 1. Asterisk symbols (*) were shown as significant difference at *p* < 0.05.

To further dissect the possible mechanisms of melatonin synthesis genes-mediated ROS accumulation and immune response, we analyzed the effects of these genes expression on the transcripts of several genes in ROS and defense pathways. Through quantitative real-time PCR, we found that these genes transient expression in tobacco leaves significantly increased the transcript levels of both ROS-related genes (*superoxide dismutase* (*SOD*), *catalase* (*CAT*) and *ascorbate peroxidase* (*APX*)) (Fig. 6) and defense-related genes (*RbohA*, *RbohB*, *pathogensis-related gene 1* (*PR1*), *PR2* and *PR5*) (Fig. 7). As common feature of defense response, ROS accumulation and PCD are involved in immune response. These results indicate the involvement of melatonin synthesis genes in both ROS and defense signalings.

**Figure 8:**
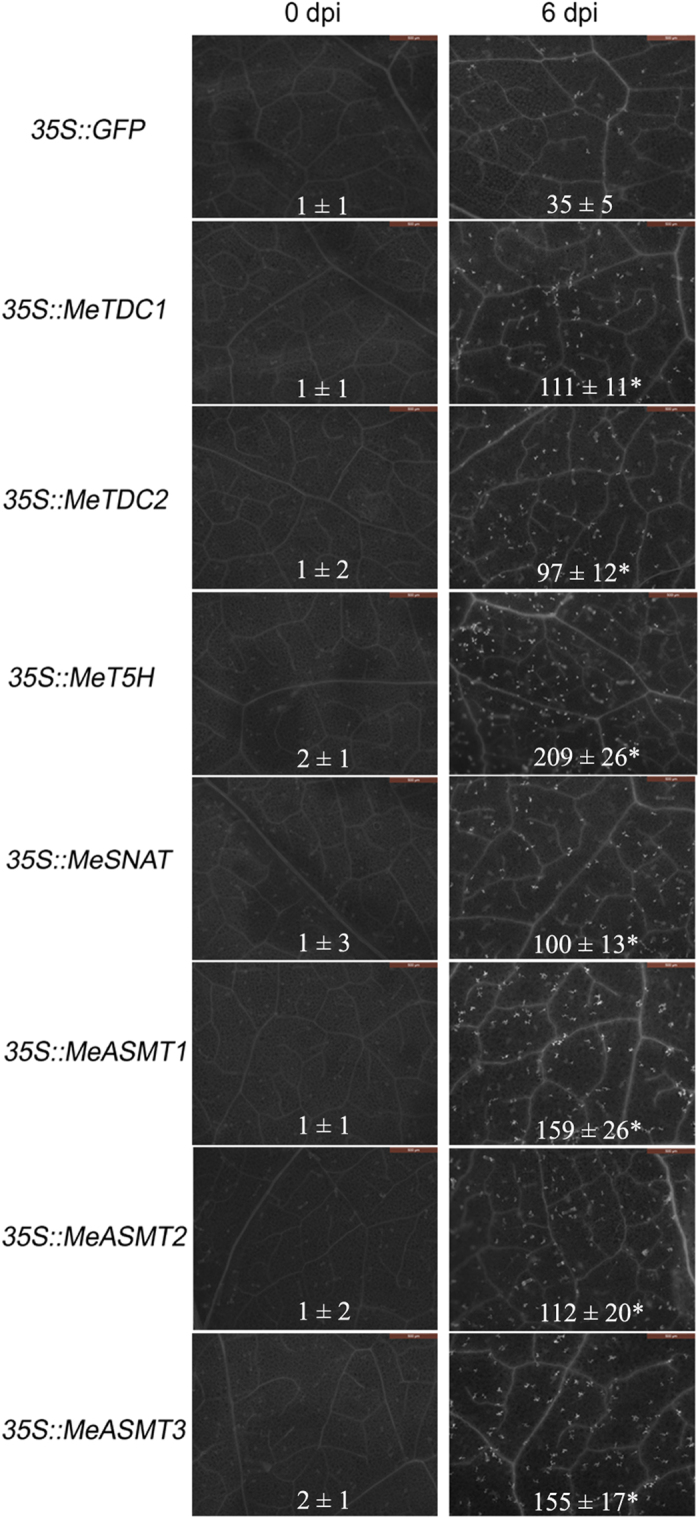
The effects of melatonin synthesis genes transient expression on callose depositions. White dots in the figures indicate callose depositions staining with aniline blue. Bar = 500 μm.

Additionally, the effects of melatonin synthesis genes transient expression on callose depositions were also analyzed. As shown in Fig. 8, the tobacco leaves expressing melatonin synthesis genes exhibited significantly more callose depositions than those from leaves expressing GFP alone. This result indicates that melatonin synthesis genes might be involved in the modulation of callose-associated cell wall and papillae-associated plant defense.

## Discussion

In the long period of evolution, plants have developed complicated mechanisms to survive and thrive in response to various environmental stresses and microbial pathogens^26^. Briefly, different stress signals are first perceived by membrane receptors, thereafter are activated by several secondary messengers such as calcium, abscisic acid (ABA), nitric oxide (NO) and H_2_O_2_. Then the signal transduction by secondary messengers leads to the activation of protein kinases, transcription factors, stress-responsive genes and physiological responses, eventually resulting in protective responses^18^,^19^,^26^,^66^,^67^,^68^,^69^,^70^. The significant elevations of endogenous melatonin in plant early stress signaling indicated that melatonin may serve as an important early messenger in plant stress response^20^,^21^,^22^,^23^,^24^,^25^,^26^,^27^.

As reviewed by Zhang *et al*.^26^, most of previous studies attribute the protective role of melatonin in stress resistance to the alleviation of stress-triggered ROS production and the activation of antioxidants. As it does in animals, melatonin has been shown to detoxify the H_2_O_2_, O_2_•^−^, singlet oxygen (^1^O_2_), hydroxyl radical (•OH), peroxynitrite anion (ONOO^−^), and hypochlorous acid (HOCl) directly^19^,^20^,^21^,^22^,^23^,^24^,^25^,^26^,^27^. Additionally, melatonin also has indirect antioxidative actions through activating the activities of antioxidant enzymes including CAT, SOD, glutathione reductase (GR), and glutathione peroxidase (GPX), all of which remove toxic reactants metabolically^19^,^20^,^21^,^22^,^23^,^24^,^25^,^26^,^27^. These actions of melatonin including potent antioxidant and radical free scavenger may largely contribute to melatonin-conferred stress resistance in plants.

Recently, our studies together with other studies provided some clues for the molecular mechanisms of melatonin-mediated stress responses in plants^18^,^19^,^26^,^62^,^63^,^64^,^65^,^66^,^67^. ROS burst and associated changes such as the transcripts of defense genes play important roles in plant immune response, especially in plant-pathogen interaction^77^,^78^. However, the involvement of melatonin in hypersensitive-like cell death and underlying ROS accumulation remain unknown. Herein, the identification and functional analysis of melatonin synthesis genes in cassava provided direct link between melatonin and immune response, as well as the underlying mechanism of these genes in programmed-like cell death and ROS accumulation. ROS is important signal molecules in signal transduction, serving as second messengers, whereas ROS overproduction under stress conditions results in serious cell damage. Interestingly, melatonin also has dual roles in regulating ROS. On one hand, stress induced melatonin relieves oxidative stress damage by decreasing excess ROS^18^,^19^,^26^,^62^,^63^,^64^,^65^,^66^,^67^. On the other hand, melatonin induces the ROS level to activate the downstream responses in the early stress response. Thus, the dual roles of melatonin in regulating ROS further indicate the protective effect of melatonin in various stress response.

In this study, we successfully identified 7 melatonin synthesis genes (2 *MeTDCs*, 1 *MeT5H*, 1 *MeSNAT* and 3 *MeASMTs*) in cassava and cloned the coding sequences of them (Fig. 1 and Table 1). The responses of these genes under different treatments (flg22, *Xam*, melatonin and H_2_O_2_) were analyzed through quantitative real-time PCR, the common expression profile of these genes indicates the possible role of them as well as melatonin in immune response and ROS signaling in cassava (Fig. 2). As a rate limited enzyme of melatonin synthesis, gene expression of MeSANT was not up-regulated by the flg22 and H_2_O_2_. However, the endogenous melatonin levels were elevated under the treatments of flg22 and H_2_O_2_, as well as the response in hypersensitive-like cell death. On one hand, the transcript level is not always consistent with enzyme activity of MeSNAT, and the post-transcriptional regulation and post-translational regulation may also result in the issue. On the other hand, MeSNAT may only responsible for part melatonin synthesis in cassava under these stress conditions, and more other rate limiting enzymes of melatonin synthesis need to be further isolated. More importantly, we identified the novel role of transient expressing melatonin synthesis genes in hypersensitive-like cell death in leaves of *N. benthamiana*, depending on ROS accumulation and endogenous melatonin (Figs 4 and 5). SOD and CAT are major antioxidant metabolic enzymes by catalysing O_2_^−.^ into H_2_O_2_ and O_2_^78^,^79^. *RbohA* and *RbohB* are also important regulators of not only H_2_O_2_ accumulation, but also plant immunity. *PR1*, *PR2* and *PR5* are widely known marker genes of innate immune response^66^,^79^,^80^. Further investigation of gene expression indicated that transient expressing melatonin synthesis genes induced the transcription of both ROS- and defense-related marker genes (Figs 6 and 7), suggesting that these genes might exert its function through ROS accumulation and PCD as well as immune response. Additionally, the tobacco leaves expressing these genes triggered the burst of callose depositions (Fig. 8), suggesting that these genes might be involved in the modulation of callose-associated cell wall and papillae-associated plant defense.

Pathogen associated molecular patterns (PAMPs)-triggered immunity (PTI) can resist the incidence of most pathogenic microbes, and plays important roles in plant immune response^77^,^78^,^79^. In this study, melatonin synthesis genes expression plays common roles in some PTI responses, including the transcript profile in response to flg22 and *Xam*, ROS accumulation, hypersensitive-like cell death, defense-related gene expression and callose deposition. HR symptoms with accumulation of ROS usually occur in biotic or abiotic stresses in plants, but it also happen as a constitutive activated protective mechanism in recent studies^79^. The induction of PTI response as well as HR by melatonin synthesis genes indicates the basal immunity triggered by them.

Taken together, this study shows the comprehensive transcripts and the putative roles of melatonin related genes as well as melatonin in immune responses in cassava. The results may also provide important candidate genes for genetic breeding to improved disease resistance of cassava.

## Methods

### Plant materials and growth conditions

In this study, cassava plants of South China 124 (SC124) variety were grown in pots with soil and vermiculite (1:1) (pH 5.7) in the green house, which was controlled under 12 h light/28 °C and 12 h dark/26 °C cycles, at the irradiance of about 120–150 μmol quanta m^−2^ s^−1^. Hoagland’s solution was watered twice every week to keep the well growth of cassava plants.

### RNA isolation and quantitative real-time PCR

Plant leaves were harvest for total RNA isolation using AxyPrep^TM^ Multisource Total RNA Miniprep Kit (AYXGEN-09113KD1, Santa Clara, California, USA) according to the manufacturer’s instruction^81^. First-strand cDNA synthesis was performed using reverse transcriptase (Thermo-K1622, Waltham, Massachusetts, USA) as Shi *et al*. described previously^19^. Thereafter, the diluted cDNA and SYBR^®^ Premix DimerEraser^TM^ (TaKaRa Biotechnology-RR091A, Dalian city, Liaoning, China) were used for quantitative real-time PCR in LightCycler^®^ 96 Real-Time PCR System (Roche, Basel, Switzerland). All gene transcripts were normalized to *elongation factor 1α* (*EF1α*) using comparative 2^−ΔΔCT^ method. The primers used in this study were listed in Supplemental Table S1.

### Plasmid construction and transient expression in tobacco leaves

The coding regions of *MeTDC1*, *MeTDC2*, *MeSNAT*, *MeT5H*, *MeASMT1*, *MeASMT2* and *MeASMT3* were amplified and cloned into downstream of GFP through restriction enzyme digestion and ligase in the pEGAD vector the control of CaMV 35S promoter^82^. The corresponding primers for gene clone were listed in Table S2. After sequencing for confirmation, the *35S::GFP-MeHSP90s* plasmids were transformed into *Agrobacterium tumefaciens* strain GV3101 and used for the subcellular localization and overexpressing analysis.

The equal volumes of GV3101 cell culture harbouring the *35S::GFP*, *35S::GFP-MeTDC1*, *35S::GFP-MeTDC2*, *35S::GFP-MeSNAT*, *35S::GFP-MeT5H*, *35S::GFP-MeASMT1*, *35S::GFP-MeASMT2* and *35S::GFP-MeASMT3* plasmids and P19 were co-infected into 30-day-old tobacco leaves as previously described^83^. For each vector construct, 20 independent leaves of five independent tobacco (*N. benthamiana*) plants (four leaves per plant) were used for GFP and physiological assays. After two days, GFP signals in the transformed tobacco leaves were observed using a confocal laser-scanning microscope (TCS SP8, Leica, Heidelberg, Germany). Physiological assays were performed at different time points after infection.

### Determination of physiological parameters

For the ROS staining, H_2_O_2_ and O_2_•^−^ were stained in diaminobenzidine (DAB) solution (pH 3.8) and nitro blue tetrazolium (NBT) solution (pH 7.8) and further decolorized in 70% (v/v) ethanol as Shi *et al*. described^19^. Moreover, H_2_O_2_ and O_2_•^−^ contents were quantified by examining the peroxide-titanium from the titanium sulphate regent and antibody-antigen-enzyme-antibody complex from the plant O_2_•^−^ ELISA Kit as Shi *et al*. described^19^.

The EL was calculated as the relative value of initial conductivity (C_i_) to the maximum conductivity (C_max_), which were assayed in the mixture of plant leaves-deionized water after 6 hr of shaken and after boiled and cooling, respectively.

The MDA was determined by examining the absorbance of the supernatant of leaves samples in the thiobarbituric acid (TBA) regent as Shi *et al*. described^19^.

### Callose staining

Tobacco leaves were harvest after infiltration GV3101 cell culture harbouring the construction, cleared in alcoholic lactophenol solution and staining with 0.01% (w/v) aniline blue as Hauck *et al*. described^84^. Images were examined using a fluorescence microscope (DM6000B, Leica, Heidelberg, Germany), and the callose depositions were quantified using the ImageJ software.

### Significant difference analysis

In this study, all experiments were repeated with three biological experiments. All data were shown as average value ± SD of three biological experiments. ANOVA and student’s *t*-test were performed to analyze the significant difference in comparison to mock treatment, and asterisk symbols (*) were shown as significant difference at *p* < 0.05.

## Additional Information

**How to cite this article**: Wei, Y. *et al*. Comprehensive transcriptional and functional analyses of melatonin synthesis genes in cassava reveal their novel role in hypersensitive-like cell death. *Sci. Rep*. **6**, 35029; doi: 10.1038/srep35029 (2016).

## Supplementary Material

Supplementary Information

## Figures and Tables

**Table 1:** The detailed information of 7 melatonin synthesis genes in cassava.

Gene	Locus Name	Gene Location	Amino acids	MW (kDa)	pI
*MeTDC1*	Manes.12G079500	Chromosome12:8842637..8844316	496	54.68	6.42
*MeTDC2*	Manes.12G038600	Chromosome12:3170788..3183555	487	53.99	5.87
*MeT5H*	Manes.06G111700	Chromosome06:22067682..22069147	438	49.71	5.89
*MeSNAT*	Manes.08G168900	Chromosome08:32904558..32908984	245	27.57	5.60
*MeASMT1*	Manes.13G140900	Chromosome13:26943587..26944856	371	41.66	5.93
*MeASMT2*	Manes.17G050500	Chromosome17:18829220..18830655	361	40.52	5.60
*MeASMT3*	Manes.13G140500	Chromosome13:26858640..26860449	362	40.67	5.61
