# Author Correction: Comprehensive transcriptional and functional analyses of melatonin synthesis genes in cassava reveal their novel role in hypersensitive-like cell death

**DOI:** 10.1038/s41598-024-66143-5

**Published:** 2024-07-12

**Authors:** Yunxie Wei, Wei Hu, Qiannan Wang, Wei Liu, Chunjie Wu, Hongqiu Zeng, Yu Yan, Xiaolin Li, Chaozu He, Haitao Shi

**Affiliations:** 1 Hainan Key Laboratory for Sustainable Utilization of Tropical Bioresources, College of Agriculture, Hainan University, Haikou, 570228 China; 2 Key Laboratory of Biology and Genetic Resources of Tropical Crops, Institute of Tropical Bioscience and Biotechnology, Chinese Academy of Tropical Agricultural Sciences, Xueyuan Road 4, Haikou, 571101 Hainan Province China

Correction to: *Scientific Reports* 10.1038/srep35029, published online 14 October 2016

This Article contains errors in Figures 4 and 8.

In Figure 4, 0 dpi-MeT5H (Figure 4A), 3 dpi-MeASMT3 (Figure 4B), 0 dpi-MeASMT2, 0 dpi-MeASMT3 and 3 dpi-MeASMT1 (Figure 4C) are incorrect. In Figure 8, 6 dpi-MeTDC1 and 6 dpi-MeASMT1 are incorrect.

The correct Figures 4 and 8 and accompanying legends appear below.

**Figure 4:**
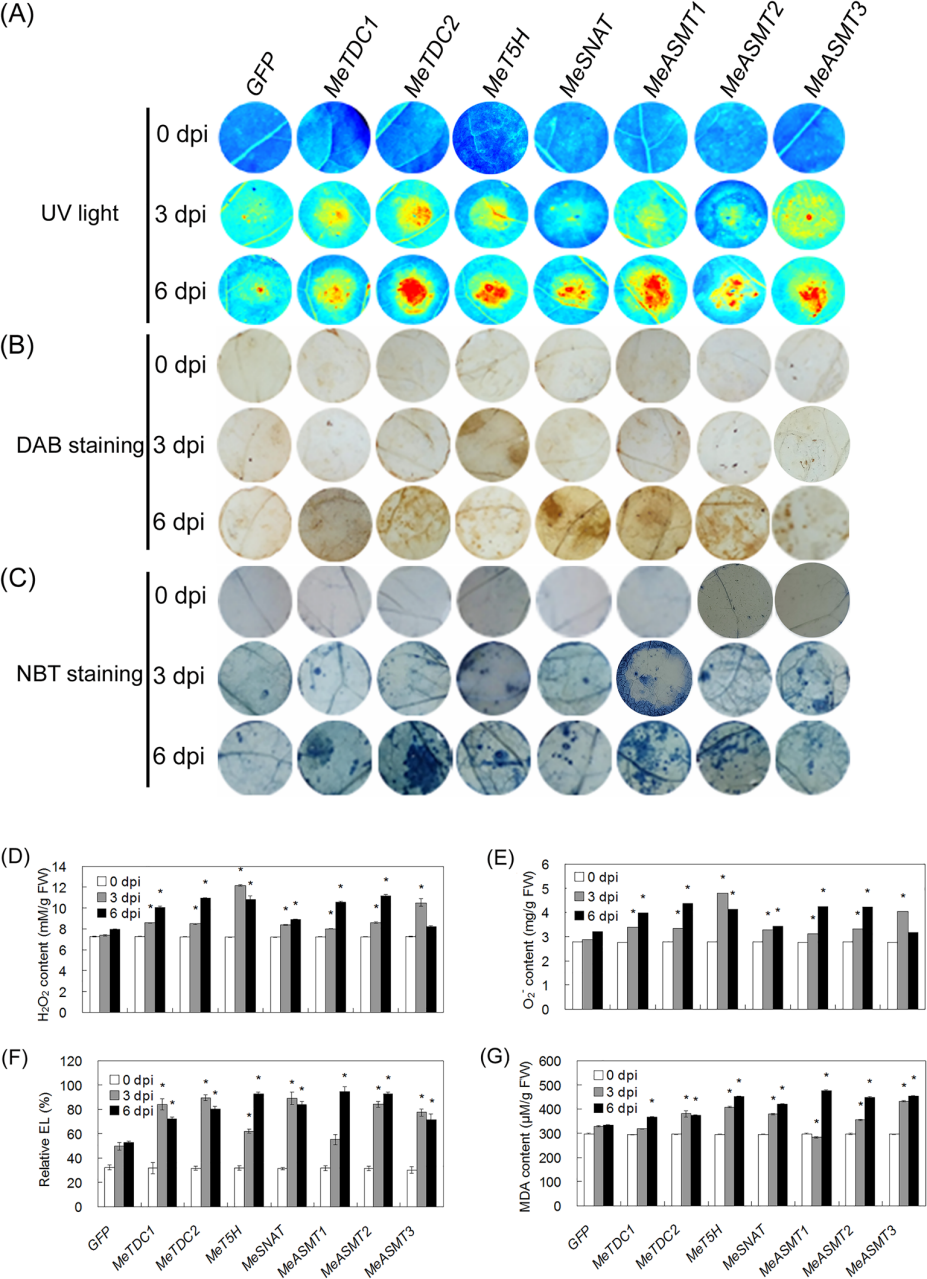
Transient expression of melatonin synthesis genes in tobacco leaves on hypersensitive response-like cell death. (**A**) Symptoms of cell death in leaves were visualized under UV light using the ChemiDoc Imaging System. (**B,C**) DAB staining for endogenous H2O2 level (**B**) and NBT staining for endogenous O2^•−^ level (**C**) in tobacco leaf discs expressing different plasmids. (**D–G**) Quantification of H2O2 (**D**), O2^•−^ (**E**), EL (**F**) and MDA (**G**) contents in leaf discs expressing different plasmids. Asterisk symbols (*) were shown as significant difference at p < 0.05.

**Figure 8:**
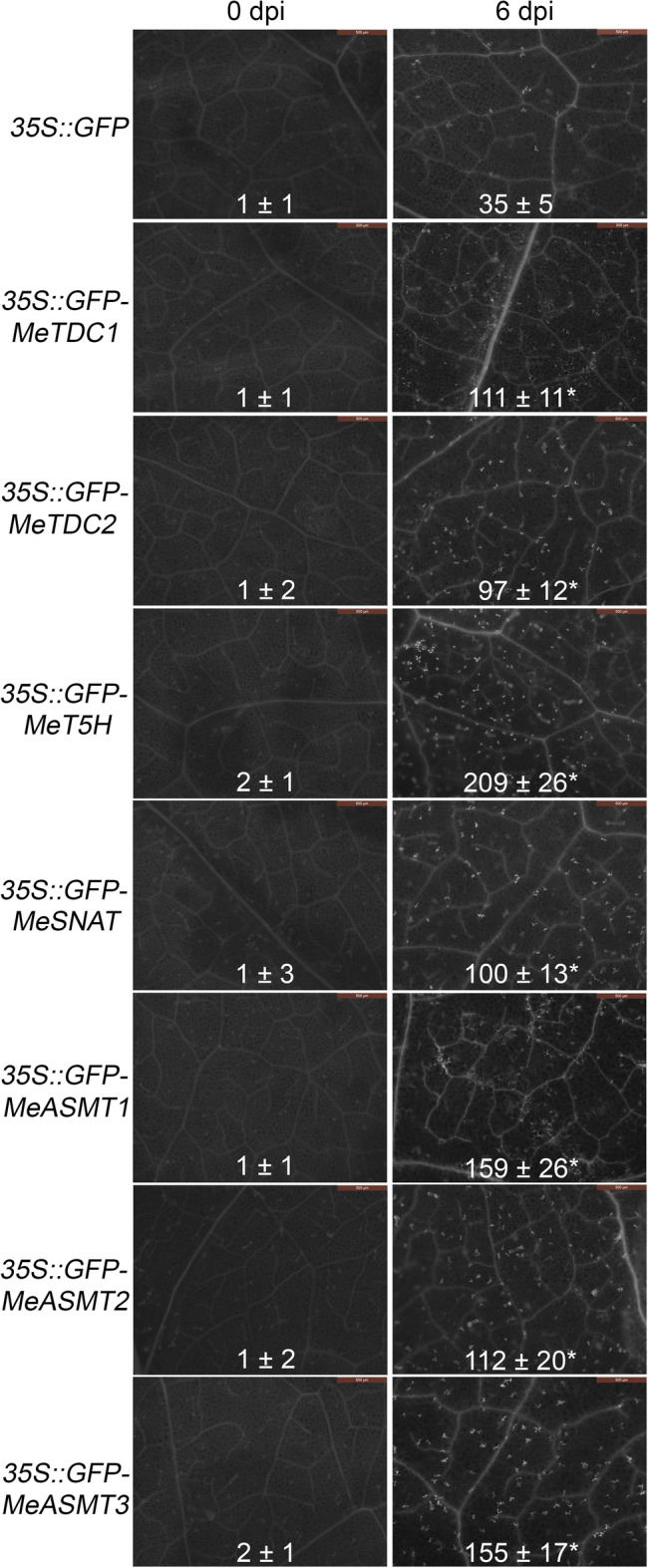
The effects of melatonin synthesis genes transient expression on callose depositions. White dots in the figures indicate callose depositions staining with aniline blue. Bar = 500 μm.

