# Supplementary material for: Comprehensive transcriptional and functional analyses of melatonin synthesis genes in cassava reveal their novel role in hypersensitive-like cell death
Source: Sci Rep. 2016 Oct 14;6:35029. doi: 10.1038/srep35029 (PMC5064325; doi:10.1038/srep35029)
Supplement: Supplementary Information [file srep35029-s1.pdf]

Comprehensive transcriptional and functional analyses of melatonin synthesis genes in cassava reveal their novel role in hypersensitive-like cell death

Yunxie Wei[1,#], Wei Hu[2,#], Qiannan Wang[1,#], Wei Liu[1], Chunjie Wu[1], Hongqiu Zeng[1], Yu Yan[1], Xiaolin Li[1], Chaozu He[1,*], Haitao Shi[1,*]

[1] Hainan Key Laboratory for Sustainable Utilization of Tropical Bioresources, College of Agriculture, Hainan University, Haikou, 570228, China

[2] Key Laboratory of Biology and Genetic Resources of Tropical Crops, Institute of Tropical Bioscience and Biotechnology, Chinese Academy of Tropical Agricultural Sciences, Xueyuan Road 4, Haikou, Hainan province, 571101, China

[#] These authors contributed equally to this work.

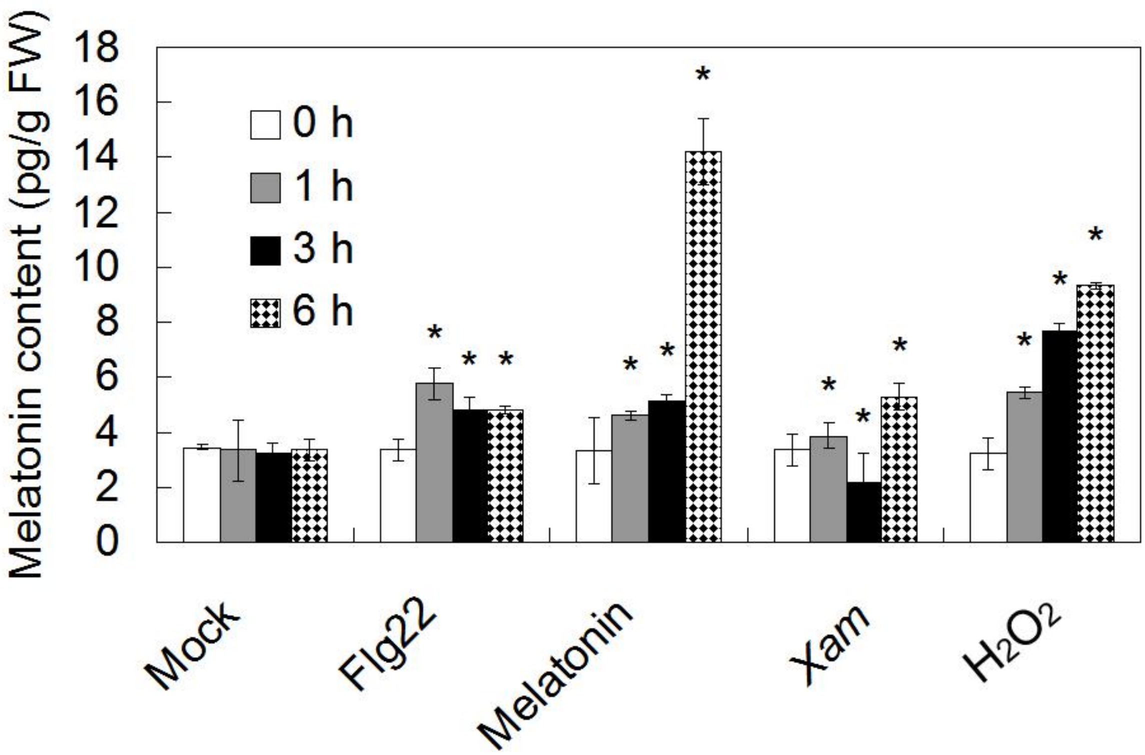

Supplementary Figure S1. The endogenous melatonin levels in response to flg22, *Xam*, melatonin and H_2O_2 treatment. For the assays, about 30-day-old cassava leaves were treated with water (mock), or 200 µM melatonin, or 10 µM flg22, or *Xam* infection, or 5 mM H_2O_2 for 0, 1, 3 and 6 h. Asterisk symbols (*) were shown as significant difference at $p<0.05$.

Supplementary Table S1. The primers used for quantitative real-time PCR.

Gene	Primer	Sequence
MeTDC1	QMeTDC1F	AGCACTGCAGCTTTTGTTGG
	QMeTDC1R	TTGAAGCAGGTGAAGCAAGC
MeTDC2	QMeTDC2F	AGTGTTGCCGGATTTTTGGG
	QMeTDC2R	TTCAAGCTCTGTTGCAGCAG
MeT5H	QMeT5HF	TCGGCGGAATTTGTGATTGC
	QMeT5HR	ATTCTCAGGAGCAGCACCAC
MeSNAT	QMeSNATF	AAGAAGCTGATTGGCATGGC
	QMeSNATR	CAATAAGAGCCTTGCCAAGACC
MeASMT1	QMeASMT1F	AATGCGCAGTTGAGCTTCAC
	QMeASMT1R	TGTGGTGGGATTTTGATGGG
MeASMT2	QMeASMT2F	CCGACAAAAGTGAACTCTCTGC
	QMeASMT2R	TTGGCTGAAGCAAAGAAGCC
MeASMT3	QMeASMT3F	TGTTTGGAGAGCCACTTTGG
	QMeASMT3R	ACTTGACAACCACTGCCTTG
NtEF1a	QNtEF1aF	AGAGGCCCTCAGACAAAC
	QNtEF1AR	TAGGTCCAAAGGTCACAA
NtRbohA	QNtRbohAF	ACACACGCCATCAGAACTCCA
	QNtRbohAR	CCCACCCAACCAAAATACGC
NtRbohB	QNtRbohBF	GTTTGCCAGCCACCACCTAAT
	QNtRbohBR	AAGAGCAGAACGAGCATCACC
NtPR1	QNtPR1F	CCGTTGAGATGTGGGTCAAT
	QNtPR1R	CGCCAAACCACCTGAGTATAG
NtPR2	QNtPR2F	CAACCCGCCCAAAGATAGTA
	QNtPR2R	TGGCTAAGAGTGGAAGGTTATG
NtPR5	QNtPR5F	GCTCGATTACGTCTTGTCTCTC
	QNtPR5R	CTCTAGCATGGTGGATTGACTT
NtSOD	QNtSODF	GCCGTCCTTAGCAGCAGTGAA

	QNtSODR	CCGGGTTTTAGGCCAGAGACAT
NtCAT	QNtCATF	CACAGCCACGCTACTCAAGAC
	QNtCATR	CCACCCACCGACGAATAAAG
NtAPX	QNtAPXF	CGCTCCTCTTATGCTCCGTCTT
	QNtAPXR	GGTGGCTCTGTCTTGTCCTCTC

Supplementary Table S2. The primers used for vector construction.

Gene	Primer	Sequence
MeTDC1	MeTDC1F	TCCCCCCGGGATGGGTAGCCTGAGTCCAAA
	MeTDC1R	CGGGATCCTTAAGGACAGACTGCATCAG
MeTDC2	MeTDC2F	TCCCCCCGGGATGGAAGGGGAGTTGAGACC
	MeTDC2R	CGGGATCCTCATAGACTCCCTAGCAAAG
MeT5H	MeT5HF	TCCCCCCGGGATGCTCTTGCAATTCGGGCA
	MeT5HR	CGGGATCCTCAAGGATGGTACAACGTAG
MeSNAT	MeSNATF	TCCCCCCGGGATGCTAACGCAGAACTTGAA
	MeSNATR	CGGGATCCCTAATACTTTGGGTACCAAA
MeASMT1	MeASMT1F	TCCCCCCGGGATGTGTAGCAGCAAGAACAA
	MeASMT1R	CGGGATCCTCAAGCATAAACCTCAATGA
MeASMT2	MeASMT2F	TCCCCCCGGGATGGAGTTACATCAAGAGCT
	MeASMT2R	CGGGATCCCTATGGATAAACCTCAATGA
MeASMT3	MeASMT3F	TCCCCCCGGGATGGAGTTGGTCAGCGGAGA
	MeASMT3R	CGGGATCCTTAAGGATAAACCTCAATGA
